# The discriminatory power of visceral adipose tissue area vs anthropometric measures as a diagnostic marker for metabolic syndrome in South African women

**DOI:** 10.1186/s13098-019-0483-1

**Published:** 2019-11-08

**Authors:** Florence E. Davidson, Tandi E. Matsha, Rajiv T. Erasmus, Andre Pascal Kengne, Julia H. Goedecke

**Affiliations:** 1 Department of Medical Imaging and Therapeutic Sciences, Faculty of Health and Wellness Sciences, Cape Peninsula University of Technology, Symphony Way, Bellville, Cape Town, 7530 South Africa; 2 Department of Biomedical Sciences, Faculty of Health and Wellness Sciences, South African Medical Research Council/Cape Peninsula University of Technology/Cardiometabolic Health Research Unit, Bellville, Cape Town, 7530 South Africa; 3 Division of Chemical Pathology, Faculty of Medicine and Health Sciences, National Health Laboratory Service (NHLS), University of Stellenbosch, Cape Town, South Africa; 4 Non-Communicable Diseases Research Unit, South African Medical Research Council, Francie van Zijl Drive, Parow Valley, Cape Town, 7505 South Africa

**Keywords:** Visceral adiposity, Dual x-ray absorptiometry, Anthropometry, Metabolic syndrome

## Abstract

**Background:**

A number of studies have shown central adiposity, in particular visceral adipose tissue (VAT) accumulation to be a hallmark of metabolic syndrome (MetS). In clinical practice, waist circumference (WC) is used as a proxy for VAT.

**Aim:**

To compare the ability of dual energy x-ray absorptiometry (DXA)-derived VAT area and anthropometric measures of adiposity for diagnosing MetS in a sample of high risk South African women.

**Methods:**

MetS was quantified using the Joint Interim Statement (JIS) criteria. Fasting glucose, insulin and lipid profile were measured in 204 post-menopausal women. Anthropometry measures included body mass index (BMI), WC, waist-to-hip ratio (WHR), waist-to-height ratio (WHtR) and a body shape index (ABSI). The area under the curve (AUC) was used to assess their performance in detecting any two components of MetS (excluding WC). Optimal WC and VAT area cut-points were derived to compare their performance for diagnosing MetS and to compare to internationally recognised cut-points.

**Results:**

The highest AUC for the prediction of MetS was recorded for VAT, followed by WHtR and WC (AUC, 0.767, 0.747 and 0.738 respectively), but these did not differ significantly (all p ≥ 0.192). In contrast, VAT was significantly better than BMI (p = 0.028), hip (p = 0.0004) and ABSI (p < 0.0001). The optimal WC (94.4 cm) and VAT area (174 cm^2^ based on the Youden’s index method and 175.50 cm^2^ based on the CTL approach) cut-points performed similarly in detecting MetS.

**Conclusion:**

DXA-derived VAT and WC had the same overall performance in discriminating the presence of any 2 MetS components in high risk South African women. These findings support the current recommendations of using WC rather than VAT for MetS risk screening, as it is cheap, accessible and easy to measure.

## Introduction

The International Diabetes Federation estimates the global prevalence of MetS to be around 25% [1, 2]. Previous research in South Africa showed that the mixed ancestry population were a high risk group for MetS [3]. It is therefore essential in a high risk population like this that one is able to identify those at risk and introduce lifestyle modifications.

Typically WC is the accepted proxy of visceral adipose tissue (VAT) and measure of central adiposity, and is used in the clinical diagnosis of MetS [4]. The advantage of WC is that it is quick and easy to measure and does not require technical equipment [5, 6]. Other anthropometric measures of total adiposity and body fat distribution such as BMI, WC, hip circumference (HC), waist to height ratio (WHtR), waist to hip ratio (WHR) and more recently, a body shape index (ABSI) have also been used as indicators of cardiometabolic risk [7]. All have their benefits, as well as their weaknesses. For example, while BMI is most commonly used as an indicator of total adiposity, it does not differentiate between muscle and fat or the location of fat [8]. WHtR, unlike WC thresholds, takes into account body size [8] and has shown closer agreement of values between men and women at all ages [9]. Similarly, the ABSI takes into account body size and is based on the WC adjusted for height and weight [10]. In contrast, WHR, uses the ratio of WC and HC, however the practicality of measuring 2 circumferences may be cumbersome and prone to error [11].

While metabolic abnormalities can be due to differential distribution of adipose tissue and or adipose dysfunction [12], a major limitation of WC, and other anthropometric measurements, is the inability to discriminate between VAT and subcutaneous adipose tissue (SAT) [6, 13]. Findings of the Framingham heart study revealed that both SAT and VAT correlated with metabolic risk factors, but that VAT was more powerfully associated with an unfavourable metabolic risk profile even after accounting for easily measured anthropometric indexes such as BMI and WC [14]. The mechanisms linking VAT accumulation to metabolic complications involve the greater production of proinflammatory cytokines and the greater lipolytic action compared to SAT, with the resultant increase in cytokines and free fatty acid transfer to the hepatic portal system impacting on insulin sensitivity [15].

In the clinical setting, VAT is however difficult to measure as it requires expensive technical equipment for imaging [6, 13, 16]. Although computed tomography (CT) and magnetic resonance imaging (MRI) are considered the gold standard imaging methods for quantifying body fat and its distribution, reliable algorithms have been developed using dual x-ray absorptiometry (DXA) software. These have been validated against CT in women with varying body mass index (BMI) [17, 18]. Numerous studies in various populations have identified associations between DXA-derived VAT area and cardio-metabolic risk factors [19–22]. Although waist circumferences may be similar, ethnic variations in VAT and subcutaneous (SAT) have been documented amongst different ethnic groups [15]. Few studies have attempted to measure VAT in African populations and more specifically in the mixed ancestry women of South Africa where the prevalence of MetS is high.

The main aim of the study was to compare the ability of DXA-derived VAT area and anthropometry measures of total and central adiposity for diagnosing MetS in this high-risk sample of South African women.

## Materials and methods

### Study setting and population

#### Participants

The current study data was collected from participants from the Cape Town Vascular and Metabolic Health (VMH) study, an extension of the Cape Town Bellville South study described previously [23, 24]. A mixed race was chosen as this population accounts for 8.9% of the South African population, 48.8% of the population of the Western Cape Province and 76% of the geographical area surveyed. This population has a high prevalence of MetS and type-2 diabetes [3], and therefore risk detection is key to early prevention and management.

Self-described mixed-ancestry female volunteers who took part in the above mentioned cross-sectional study were invited to complete a whole body DXA scan. The DXA scans were performed from April 2015 to June 2016. Being 20 years or older was an inclusion criterion. Subjects were excluded if they were pregnant or acutely ill. Ethical approval was obtained from the Ethics Committees of the Cape Peninsula University of Technology and Stellenbosch University (NHREC: REC-230 408-014, CPUT/HWS-REC 2015/H03 and N14/01/003). Participants provided written consent to participate in the study. A total of 204 women volunteered for the study.

#### Body composition

Anthropometric measurements were taken, as described in detail previously [3, 24]. Body weight was measured (to the nearest 0.1 kg) with the participants in light clothing and without shoes. Height was measured to the nearest centimetre using a stadiometer. BMI was calculated as weight divided by height in square meters (kg/m^2^). Waist circumference was measured using a non-elastic tape at the level of the narrowest part of the torso as seen in an antero-posterior projection and hip circumference around the widest part of the buttocks. The WHtR is calculated as the waist measurement divided by the height, while WHR is calculated as waist measurement divided by the hip measurement. ABSI was based on WC adjusted for weight and height, and was calculated using the formula, ${\text{ABSI}} = \frac{WC}{{BMI^{2/3 } height^{1/2} }}$ [10]. All anthropometric measurements were performed three times, and the average measurements were used for analysis.

Body composition (fat mass and fat-free mass) was acquired by a trained and experienced radiographer using a Hologic Discovery W DXA whole body scanner configured with software version 13.4.1 (Hologic, Bedford, MA). Participants were positioned as per the NHANES body composition manual as advocated by Hangartner [25]. VAT and SAT area were estimated within the android region, which is automatically defined with a caudal limit placed on top of the iliac crests and its height set to 20% of the distance from the top of the iliac crest to the base of the skull as the cephalic limit [18]. DXA has proved to be as accurate as a clinical CT scan in the quantification of VAT and SAT in adults [18]. Subtotal body fat % and kg, which excludes the head, were used in the analysis. The head was excluded to reduce the possibility of any artefacts in the head region. Total adipose body tissue classification excludes the head.

#### Blood sample collection and analysis

After an overnight fast, blood samples were taken to measure glycated haemoglobin (HbA_1c_), glucose, insulin and lipid profile. Blood samples were transported daily on ice for processing using standard pathology practices. Biochemical parameters were analysed at an ISO 15189 accredited Pathology practice (Pathcare, Reference Laboratory, Cape Town, South Africa) as described elsewhere [24]. Plasma glucose was measured by the enzymatic hexokinase method (Beckman AU, Beckman Coulter, South Africa). Insulin was measured by paramagnetic particle chemiluminescence assay (Beckman DXI, Beckman Coulter, South Africa). High-density lipoprotein cholesterol (HDL-C) was by enzymatic immunoinhibition, and triglycerides by glycerol phosphate oxidase–peroxidase and Low density lipoprotein cholesterol (LDL-C) by enzymatic selective protection—End Point (Beckman AU, Beckman Coulter, South Africa).

Metabolic syndrome was quantified using the Joint Interim Statement (JIS) criteria [4], namely the presence of any 3 risk factors: WC ≥ 80 cm, elevated triglycerides ≥ 1.7 mmol/L (or drug treatment for elevated levels), elevated blood pressure systolic ≥ 130 and/or diastolic ≥ 85 mmHg (or antihypertensive drug treatment), elevated fasting blood glucose ≥ 5.6 mmol/L (or drug treatment of elevated glucose) and reduced HDL-C < 1.3 mmol/L (or drug treatment for reduced HDL-C). In our analysis we excluded WC in the discrimination of MetS as WC is part of the JIS MetS criteria, but used DXA-VAT as an independent variable to discriminate those with MetS.

### Statistical methods

General characteristics of the study groups are summarized as count and percentage for categorical variables, mean and standard deviation (SD) or median and 25th–75th percentiles for quantitative variables. The *pROC* package [26] of the R statistical software version 3.4.3 (30 Nov 2017), (The R Foundation for Statistical Computing, Vienna, Austria) was used for receiver operating characteristics (ROC) analyses. The area under the curve (AUC) was then used to assess and compare the ability of VAT area, WC, BMI, WHR, WHtR and ABSI to predict the presence of any two components of metabolic syndrome, excluding WC, with AUC comparisons through non-parametric methods [27]. The optimal WC and VAT area was determined by applying both the Youden’s index approach [28] and the closest top left point approach [29]. For comparison purposes, the optimal VAT and WC thresholds derived for this sample were tested alongside cut points commonly advocated in African and other populations [4, 30, 31].

## Results

### Participant characteristics

The characteristics of the participants are presented in Table 1. The mean age of the participants was 53.1 (± 13.7) years. The mean BMI was 32.6 (± 7.2), with 19.8% of the participants being overweight, and the majority (64.7%) obese. Twenty seven percent were classified as having diabetes and 57.1% had MetS, with 88.7% presenting with a WC ≥ 80 cm. The median VAT area was 181 cm^2^. The most prevalent MetS components after the high WC, was high blood pressure (74%), low HDL (48%) with triglycerides (34.3%) and high fasting glucose (33.5%) being the least frequent component.

**Table 1:** Participant characteristics

Variable	Overall
n	204
Age (years)	53.1 (13.7)
Body composition	
Height (m)	1.56 (0.06)
Weight (kg)	79.3 (18.3)
BMI (kg/m^2^)	32.6 (7.2)
WC (cm)	99 (15.0)
Hip (cm)	113 (14.0)
WHR	0.88 (0.07)
WHtR	0.64 (0.1)
ABSI	0.078 [0.075–0.082]
Body fat (%)	44.0 (39.8–48.6)
Body fat (kg)	31.2 (24.4–40.0)
VAT area (cm^2^)	181.2 [134.7–235.0]
Cardiometabolic risk factors	
Fasting glucose (mmol/L)	6.3 (3.3)
Fasting serum insulin (µU/mL)	8.4 (5.7–12.7)
Triglycerides (mmol/L)	1.6 (1.0)
LDL-cholesterol (mmol/L)	3.3 (1.0)
HDL-cholesterol (mmol/L)	1.3 (0.3)
Total cholesterol (mmol/L)	5.4 (1.2)
Systolic blood pressure (mmHg)	127 (21)
Diastolic blood pressure (mmHg)	82 (12)
Metabolic syndrome (JIS)	
High waist circumference (n(%))	181 (88.7)
High blood pressure (n(%))	151 (74.0)
High fasting blood glucose (n(%))	68 (33.5)
High triglycerides (n(%))	70 (34.3)
Low HDL (n(%))	98 (48.0)
3 components or more (n(%))	116 (57.1)

Values are mean (standard deviation) or median (interquartile range)

*BMI* (WHO classification) body mass index, *WC* waist circumference, *WHR* waist- to- hip ratio, *WHtR* waist- to -height-ratio, *ABSI* A Body Shape Index, *VAT* visceral adipose tissue

### Discriminatory power of anthropometric variables and VAT for the prediction of metabolic syndrome

The discriminatory power of all anthropometric variables and VAT area for the prediction of any two JIS-defined components of MetS (excluding WC, as this is part of the MetS criteria) is shown in Table 2. The highest point estimate of AUC for the prediction of MetS was recorded for VAT, followed by WHtR and WC (AUC, 0.767, 0.747 and 0.738 respectively), but these did not differ significantly (all p ≥ 0.192). In contrast, VAT had significantly greater discriminatory power than BMI (p = 0.028), and VAT, WHtR and WC had greater discriminatory power than hip (p < 0.0004) and ABSI (p < 0.0001).

**Table 2:** Comparison of the performance of anthropometric variables and VAT area in the discrimination of any two components of metabolic syndrome (not including the WC criteria in the analysis)

Variables	AUC (95% CI)	*p* value for differences in AUC						
		vs BMI	vs WC	vs Hip	vs WHR	vs WHtR	vs ABSI	vs VAT area
BMI	0.716 (0.643–0.788)	–	0.187	0.002	0.703	0.109	0.014	0.028
WC	0.738 (0.667–0.810)	0.187	–	0.0005	0.288	0.413	0.0004	0.192
Hip	0.664 (0.587–0.741)	0.002	0.0005	–	0.528	0.001	0.132	0.0004
WHR	0.698 (0.624–0.771)	0.703	0.288	0.528	–	0.181	< 0.0001	0.083
WHtR	0.747 (0.675–0.819)	0.109	0.413	0.001	0.181	–	0.0001	0.397
ABSI	0.575 (0.495–0.655)	0.014	0.0004	0.132	< 0.0001	0.0001	–	< 0.0001
VAT area	0.767 (0.700–0.834)	0.028	0.192	0.0004	0.083	0.397	< 0.0001	–

*ABSI* A Body Shape Index, *AUC* area under the receiver-operating characteristic curve, *BMI* body mass index, *Hip* hip circumference, *WC* waist circumference, *WHR* waist-to-hip ratio, *WHtR* waist-to-height ratio, *95% CI* 95% confidence interval

### Optimal waist circumference and VAT threshold values

**Fig. 1:**
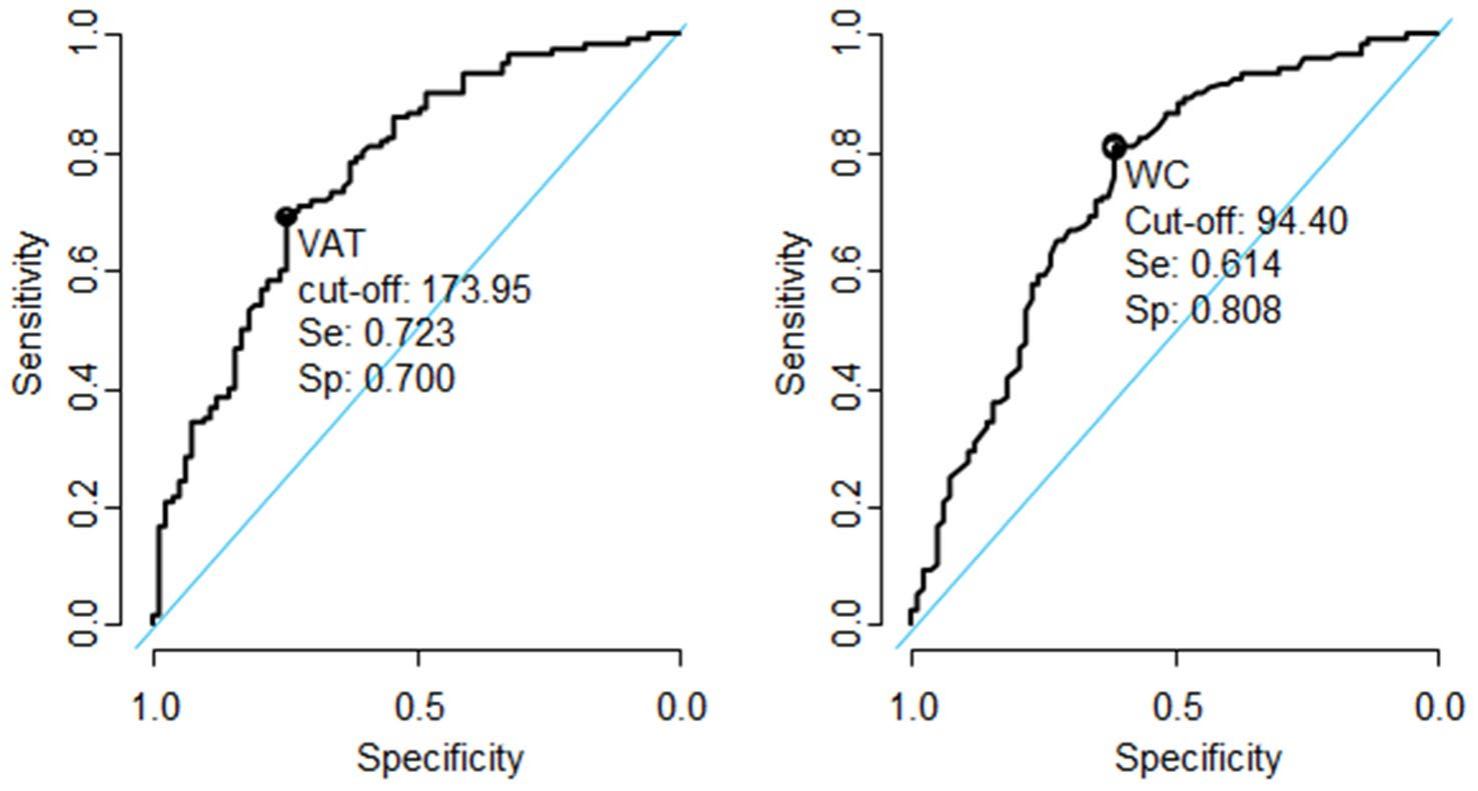
Receive operating characteristic curves (ROC) using visceral adipose tissue area (VAT) and waist circumference (WC) for the prediction of the presence of at least two components of the metabolic syndrome. *Se* sensitivity, *Sp* specificity (using the Youden Index)

Given that WC is commonly used as a proxy for VAT in the clinical setting, we examined the thresholds for WC and VAT area to diagnose MetS (excluding WC) in this sample. The receiver operating characteristic curves (ROC) for the prediction of the presence of at least two components of MetS using WC or VAT area are presented in Fig. 1. The Youden’s index method and the CTL approach used to derive the optimal WC threshold values in this sample identified the same threshold of 94.4 cm (Table 3). The optimal VAT area threshold was 174 cm^2^ based on the Youden’s index method and 175.50 cm^2^ based on the CTL approach.

**Table 3:** Performance of different VAT and waist optimal thresholds to detect two components of the metabolic syndrome (excluding waist circumference)

Thresholds^a^	Methods	App prev	True prev	Sensitivity	Specificity	Accuracy	DOR	NND	Youden index	PPV	NPV	LR+	LR−
VAT area													
174.0 (137.7–181.5)	Youden index	47.3 (40.3–54.4)	40.9 (34.0–48.0)	0.723 (0.614–0.815)	0.700 (0.610–0.780)	0.709 (0.642–0.770)	6.02 (3.28–11.31)	2.36 (1.68–4.48)	0.423 (0.223–0.596)	0.625 (0.520–0.722)	0.785 (0.695–0.859)	2.41 (1.78–3.27)	0.40 (0.27–0.57)
175.50 (154.6–180.1)	CTL	47.8 (40.7–54.9)	40.9 (34.0–48.0)	0.735 (0.627–0.826)	0.700 (0.610–0.780)	0.714 (0.647–0.775)	6.47 (3.46–12.08)	2.30 (1.65–4.23)	0.435 (0.236–0.606)	0.629 (0.525–0.725)	0.792 (0.703–0.865)	2.45 (1.81–3.31)	0.38 (0.26–0.55)
WC													
94.40 (89.7–102.3)	Youden index	36.4 (29.8–43.5)	40.9 (34.0–48.0)	0.614 (0.501–0.719)	0.808 (0.726–0.874)	0.729 (0.662–0.789)	6.72 (3.56–12.67)	2.36 (1.68–4.39)	0.423 (0.228–0.594)	0.689 (0.571–0.792)	0.752 (0.668–0.824)	3.21 (2.14–4.81)	0.48 (0.36–0.63)
94.40 (93.3–101.2)	CTL	36.4 (29.8–43.5)	40.9 (34.0–48.0)	0.614 (0.501–0.719)	0.808 (0.726–0.874)	0.729 (0.662–0.789)	6.72 (3.56–12.67)	2.36 (1.68–4.39)	0.423 (0.228–0.594)	0.689 (0.571–0.792)	0.752 (0.668–0.824)	3.21 (2.14–4.81)	0.48 (0.36–0.63)
80^b^		10.8 (6.9–15.9)	40.9 (34.0–48.0)	0.205 (0.124–0.307)	0.958 (0.905––0.986)	0.650 (0.580–0.716)	5.92 (2.09–16.79)	6.13 (3.40–33.87)	0.163 (0.029–0.294)	0.773 (0.546–0.922)	0.635 (0.561–0.705)	4.92 (1.89–12.80)	0.83 (0.74–0.93)
90^b^		26.1 (20.2–32.7)	40.9 (34.0–48.0)	0.482 (0.371–0.594)	0.892 (0.822–0.941)	0.724 (0.657–0.784)	7.66 (3.73–15.71)	2.68 (1.87–5.19)	0.374 (0.193–0.535)	0.755 (0.617–0.862)	0.713 (0.634–0.784)	4.45 (2.54–7.78)	0.58 (0.47–0.72)

*App prev* apparent prevalence, *DOR* diagnostic odd ratio, *LR−* likelihood of a negative test, *LR+* likelihood of a positive test, *NND* number needed to diagnose, *NPV* negative predictive value, *PPV* positive predictive value, *thresh* threshold, *True prev* true prevalence

^a^CI P2.5 and P97.5 from bootstrap, WC 80 cm^b^ (JIS), WC 90 cm^b^ [31]

The WC cut-point of 94.4 cm in this sample yielded a sensitivity of 61.4% and specificity of 80.8%. The sensitivity of this cut-point was higher than the recommended JIS ≥ 80 cm WC (20.5%) cut-point, and the 90 cm WC (48.2%) cut-point recommended by Matsha and co-workers for both genders in the larger study of this population [31]. Conversely, the specificities of the WC derived for this sample (80.8%) was lower than that for the JIS criteria (95.8%) and the larger study in this population (89.2%) [31]. The sensitivity and specificity of the 174 cm^2^ VAT thresholds obtained from the Youden index were 72.3% and 70% respectively.

When comparing the sensitivity and specificity of WC and VAT cut-points for predicting any two components of MetS in this population, the sensitivity of the VAT area cut-point was greater than WC (72.3% vs 61%), however the specificity of VAT area was less than WC (70% vs 80.8%). When comparing the accuracy of VAT and WC for predicting any two components of MetS, they were very similar (70.9–72.3%, for Youden index and CTL). Further, the positive predictive values (PPV) were similar for VAT area and WC in diagnosing any two components of MetS (63% vs 69%) with similar 95% CI’s. The likelihood of a positive test (LH^+^) for VAT area and WC were also similar (2.1–2.45 vs 3.21 respectively).

## Discussion

In this study we set out to compare the discriminatory power of DXA-derived VAT area and other anthropometric measurements to diagnose any two components of MetS in a sample of high-risk mixed ancestry women from South Africa. The main finding was that VAT area, WC and WHtR performed similarly, lending support to the current recommendation of using the WC measurement for the diagnosis of MetS.

VAT accumulation is an important predictor of MetS [32] and is more closely associated with MetS risk than SAT due to its greater lipolytic activity and higher inflammatory profile [15]. WC is an internationally recognized surrogate for VAT and one of the five JIS criteria for the diagnosis of MetS [4]. As WC and other anthropometric measurements are however unable to discriminate between VAT and SAT, we set out to determine whether VAT would perform better than these measures and could therefore be used in risk prediction. Notably, we found that DXA- derived VAT did not perform better that WC and some of the other measures of central adiposity in diagnosing MetS in this sample. A possible explanation for this is that abdominal SAT is heterogenous, is of greater volume than VAT and has similar metabolic activity to VAT, thus also impacting on the development of insulin resistance and MetS [12]. The larger volume of SAT vs. VAT is particularly true for women compared to men, with abdominal SAT measured at L4–L5 being roughly five fold greater than VAT in the same location [12]. Moreover, abdominal SAT can be divided at the level of the fascia superficialis into deep (dSAT) and superficial SAT (sSAT), with the dSAT having higher metabolic activity and inflammatory profile than sSAT, and intermediate to VAT [33]. This suggests that the metabolic effects of accumulation of both VAT and abdominal SAT on MetS risk may be additive. Other studies have shown that VAT area performs better than WC, WHtR and WHR in determining MetS risk. For example, the results of the cross-sectional Netherlands epidemiology of Obesity study involving mostly white middle-aged obese women indicated that MRI-derived VAT was most strongly associated with cardiometabolic risk factors followed by WC and WHR [34]. Likewise in a study in Japanese women, CT-derived VAT performed better than WC in predicting MetS [35]. In contrast, and similar to our findings, Evans et al. [36] found that WC, WHtR and a CT-derived measure of VAT performed similarly in predicting MetS in pre-menopausal black and white South African women. These findings may be explained by ethnic-specific associations between adipose tissue distribution and insulin sensitivity. Indeed, studies have shown that VAT was the most significant correlate of insulin sensitivity in white women, whereas in black women, SAT performed similarly or better to VAT [37]. Another possible explanation for VAT not performing significantly better than WC and WHtR in diagnosing MetS may lie in the methodological limitation of imaging to precisely distinguish the various anatomical adipose tissue compartments [38]. Although DXA-derived VAT and SAT have been validated against the gold standard imaging methods such as CT and MRI in other ethnic groups [17, 18], they have not yet been validated in a mixed ancestry population. Additionally, DXA is unable to differentiate between dSAT and sSAT.

When comparing the performance of the VAT area and WC thresholds for detecting any two components of MetS in this sample, we found that the sensitivity of VAT area was higher than WC (73% vs 61%), but the specificity was lower (70% vs 80.8%). The implication of this is that VAT may be more sensitive than WC to detect MetS, but may also over diagnose MetS in screening. Furthermore, the accuracy and PPV of the WC and VAT area, as well as the DOR were very similar, reiterating the view that there may not be any advantage of using the more costly measurement of VAT in the clinical setting.

In contrast to WC and WHtR, we found that VAT area performed better than BMI, HC, WHR and ABSI in diagnosing any two components of MetS. A possible reason for this is that the latter anthropometry measures are essentially measures of total adiposity, which is not as closely associated with MetS as central adiposity [7]. For example, several studies have shown weak correlations for ABSI in predicting MetS [39, 40]. Similarly in a large cross-sectional study in an Iranian population, BMI had the lowest AUC in women for predicting Mets [41]. WC only requires one measurement unlike BMI and ABSI which requires height to be measured which can be challenging in the clinical setting [42].

The optimal VAT area for predicting any 2 components of MetS (other than WC) in this sample was 174 cm^2^ (CI 137.7–181.5). This is higher than the ≥ 163 cm^2^ found in peri and post-menopausal African and Caucasian American women [19]. Our VAT thresholds were also considerably higher than those used to predict metabolic risk variables in black (> 48 cm^2^) and white (> 107 cm^2^) pre-menopausal SA women [36]. Notably, recommended VAT cut points for diagnosing any two components of MetS (other than WC) differ by age and ethnicity [19, 35, 36]. Indeed, studies have shown that for the same BMI or WC, black women have less VAT than white women [43]. Further, it is well known that VAT accumulation occurs at menopause [44], and that VAT and total fat mass are independent with regards to metabolic risk [45], supporting the notion that WC corresponding to critical levels of VAT area may be age specific [19, 35]. This is supported by results of the study by Evans et al. [36] which identified considerably lower CT-derived VAT thresholds (> 88 cm^2^) for MetS risk factors in younger pre-menopausal white South African women than those recommended for peri- and post-menopausal women from our study (174 cm^2^) and others (≥ 163 cm^2^) [19].

The strengths of the study are that we derived WC and VAT area cut-points specific to this sample, which enabled us to directly compare the performance of these cut-points to diagnose any 2 components of MetS. Additionally, the two statistical approaches used to derive the cut-points yielded very similar results, an indication that the relationships are consistent. Furthermore, this is the first study to measure DXA-derived VAT area in mixed ancestry South African women. Although CT and MRI are considered the gold-standard for measuring VAT area, DXA has been proven to be an accurate imaging tool for deriving VAT [18]. The limitations of this study are that the sample included mostly post-menopausal women and thus the results cannot be generalised to younger women or men given that there are specific age and sex thresholds for WC that correspond to critical levels of VAT cm^2^ [19, 35]. Additionally, the sample size was relatively small, yielding unstable estimates. A larger representative study is needed to determine whether VAT performs better than WC for diagnosing MetS and if shown to be better, ethnic, sex and age specific cut-points developed.

## Conclusion

Our study showed that there is no advantage of measuring VAT over WC in the diagnosis of MetS as VAT area, WC and WHtR performed similarly in predicting two components of MetS in this sample of mixed ancestry South African women. WC is easier to measure in the clinical setting than other anthropometric measures and an universally recognised proxy for central adiposity.

## Data Availability

The datasets used and/or analysed during the current study are available from the PI (TEM) Vascular and Metabolic Health Study on reasonable request.
